# Dilithium hexa­hydroxidostannate(IV) dihydrate, a second monoclinic modification with a layer structure

**DOI:** 10.1107/S1600536813008416

**Published:** 2013-04-05

**Authors:** Shouassi Kamaha, Hans Reuter

**Affiliations:** a Institut für Chemie neuer Materialien, Strukturchemie, Universität Osnabrück, Barbarastrasse 7, D-49069 Osnabrück, Germany

## Abstract

The title compound, Li_2_[Sn(OH)_6_]·2H_2_O, is dimorphic. As for the previously described α-modification, the title β-modification crystallizes in the monoclinic system and contains the same primary building units, *viz* [Sn(OH)_6_]^2−^ octa­hedra and [Li(μ_2_-OH)_3_(H_2_O)] tetra­hedra. In contrast to the Sn—O bond lengths that are very similar in both modifications, the Li—O bond lengths differ significantly, in particular those involving the water mol­ecule. In the new β-modification, the primary building units are linked into layers parallel to (010). The [Sn(OH)_6_]^2−^ octa­hedra (-1 symmetry) form hexa­gonal nets and the [Li(μ_2_-OH)_3_(H_2_O)] tetra­hedra are situated in between, with their apices in an alternating fashion up and down. O—H⋯O hydrogen bonds between OH groups and water mol­ecules exist within the layers as well as between them.

## Related literature

For background to the structures of *M*_2_[Sn(OH)_6_] with *M* = Na, K, see: Jacobs & Stahl (2000 ▶). For literature on Li_2_[Sn(OH)_6_]·*n*H_2_O, see: Reuter & Bargon (1997 ▶) for *n* = 2; Yang *et al.* (2001 ▶) for *n* = 0. For *M*[Sn(OH)_6_] compounds (*M* = divalent metal), see: Strunz & Nickel (2001 ▶); Basciano *et al.* (1998 ▶).

## Experimental

### 

#### Crystal data

Li_2_[Sn(OH)_6_]·2H_2_O
*M* *_r_* = 270.65
Monoclinic, 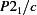

*a* = 6.1028 (2) Å
*b* = 10.4708 (3) Å
*c* = 6.0003 (2) Å
β = 118.249 (1)°
*V* = 337.76 (2) Å^3^
*Z* = 2
Mo *K*α radiation
μ = 3.78 mm^−1^
*T* = 100 K
0.15 × 0.14 × 0.11 mm

#### Data collection

Bruker APEXII CCD diffractometer
Absorption correction: multi-scan (*SADABS*; Bruker, 2009 ▶) *T*_min_ = 0.601, *T*_max_ = 0.681
11484 measured reflections
986 independent reflections
964 reflections with *I* > 2σ(*I*)
*R*_int_ = 0.024

#### Refinement

*R*[*F* ^2^ > 2σ(*F* ^2^)] = 0.013
*wR*(*F* ^2^) = 0.032
*S* = 1.09
986 reflections
54 parameters
H-atom parameters constrained
Δρ_max_ = 0.36 e Å^−3^
Δρ_min_ = −0.39 e Å^−3^

### 

Data collection: *APEX2* (Bruker, 2009 ▶); cell refinement: *SAINT* (Bruker, 2009 ▶); data reduction: *SAINT*; program(s) used to solve structure: *SHELXS97* (Sheldrick, 2008 ▶); program(s) used to refine structure: *SHELXL97* (Sheldrick, 2008 ▶); molecular graphics: *DIAMOND* (Brandenburg, 2006 ▶) and *Mercury* (Macrae *et al.*, 2008 ▶); software used to prepare material for publication: *SHELXTL* (Sheldrick, 2008 ▶).

## Supplementary Material

Crystal structure: contains datablock(s) I, global. DOI: 10.1107/S1600536813008416/wm2733sup1.cif

Structure factors: contains datablock(s) I. DOI: 10.1107/S1600536813008416/wm2733Isup2.hkl

Additional supplementary materials:  crystallographic information; 3D view; checkCIF report

## Figures and Tables

**Table 1:** Selected bond lengths (Å)

Sn1—O1	2.0567 (11)
Sn1—O3	2.0654 (11)
Sn1—O2	2.0699 (11)
O1—Li1	1.961 (3)
O2—Li1^i^	1.948 (3)
O3—Li1^ii^	1.965 (3)
Li1—O4	2.030 (3)

Symmetry codes: (i) 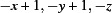; (ii) 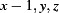.

**Table 2:** Hydrogen-bond geometry (Å, °)

*D*—H⋯*A*	*D*—H	H⋯*A*	*D*⋯*A*	*D*—H⋯*A*
O1—H1⋯O4^i^	0.96	1.84	2.7955 (16)	173
O2—H2⋯O1^iii^	0.96	1.93	2.8853 (16)	173
O3—H3⋯O4^iv^	0.96	1.90	2.8343 (16)	164
O4—H41⋯O3^v^	0.96	1.72	2.6634 (16)	167
O4—H42⋯O2^vi^	0.96	1.73	2.6662 (16)	165

Symmetry codes: (i) 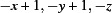; (iii) 
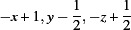
; (iv) 
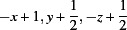
; (v) 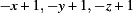; (vi) 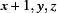.

**Fig. 1.**
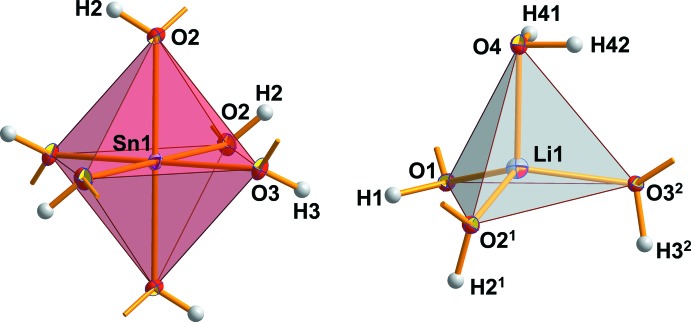
Combined polyhedra and ball-and-stick models of the coordination spheres around tin and lithium with the atomic numbering scheme used; with exception of the hydrogen atoms, which are shown as spheres with common isotropic radius, all other atoms are represented with their anisotropic displacement ellipsoids at the 50% probability level; additional bonds to neighboring metal atoms are indicated as shortened sticks.

**Fig. 2.**
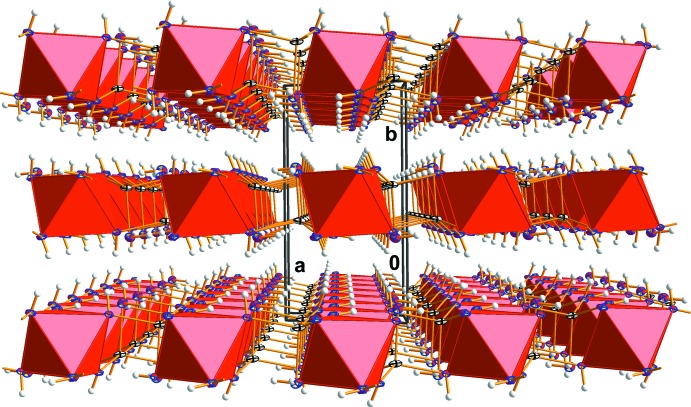
Combined polyhedra (around Sn) and ball-and-stick (around Li) model of the layered structure of the title compound viewed down [001].

**Fig. 3.**
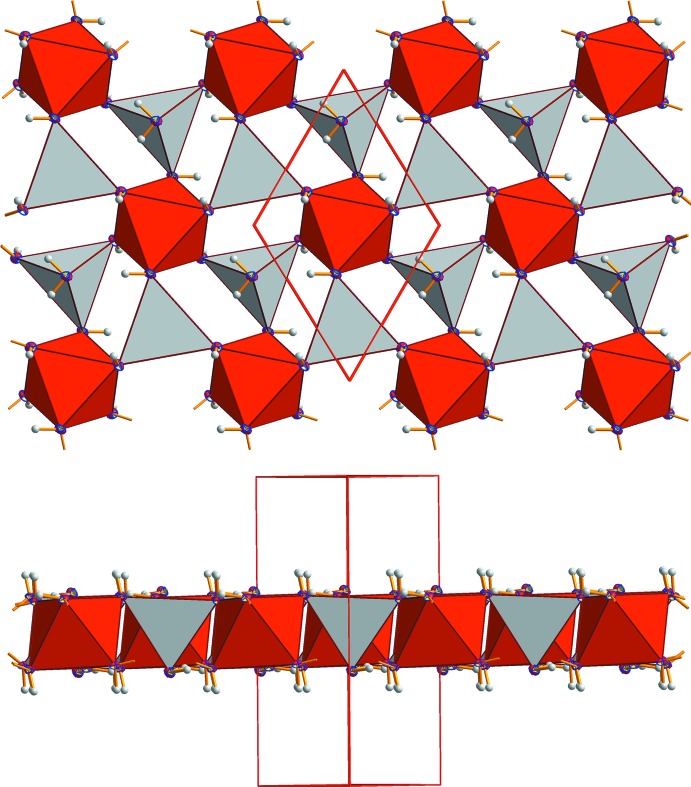
Arrangement of [Sn(OH)6] octahedra and [Li(OH)3(H2O)] tetrahedra within the almost planar layers of the title compound; top view looking down the b-axes at the head, side view looking down [110] at the bottom.

**Fig. 4.**
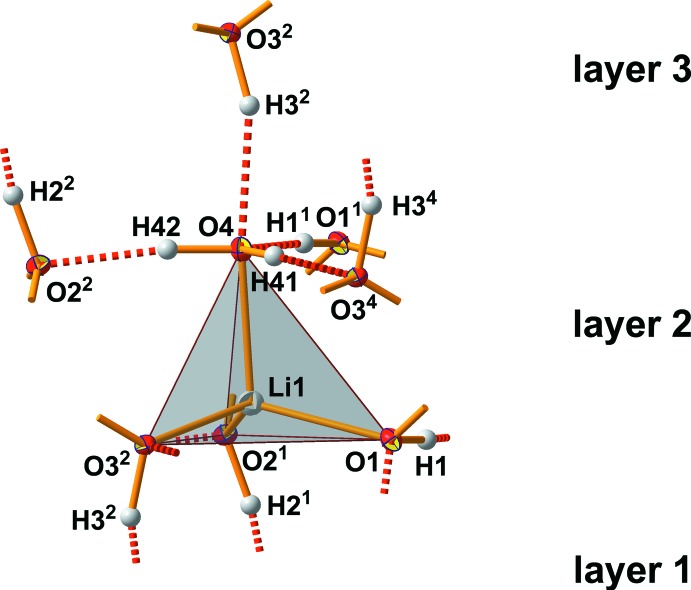
Details of the hydrogen bonding system in the crystal structure of the title compound the hydrogen and oxygen atoms of the [Li(OH)3(H2O)]-tetrahedron are involved in; with exception of the hydrogen atoms, which are shown as spheres with common isotropic radius, all other atoms are represented with their anisotropic displacement ellipsoids at the 50% probability level; hydrogen bonds indicated as red dashed, additional bonds as shortened sticks. [Symmetry codes: (1) 1 - x,1 - y, -z; (2) 1 + x, y, z; (3) 1 - x, -0.5 + y, 0.5 - z; (4) 1 - x, 1 - y, 1 - z.]

## supplementary crystallographic information

### Comment

Compounds of the hexahydroxidostannate(IV) anion, [Sn(OH)_6_]^2-^, are well defined in chemistry as well as in mineralogy. In combination with bivalent cations, the anion is found in some rare tin minerals of natural and anthropogenic (Basciano *et al.*, 1998) origin like schoenfliesite, Mg[Sn(OH)]_6_, the prototype and name giver for a large group of isostructural compounds of composition *M*[Sn(OH)_6_] with *M* = Mg, Fe, Mn, Zn and Ca (Strunz & Nickel, 2001). Corresponding compounds of univalent cations are of general formula *M*_2_[Sn(OH)]_6_ or exist as hydrates, *M*_2_[Sn(OH)_6_]^.^*n*H_2_O. The anhydrous compounds of sodium and potassium are long known and commercially available products (preparing salt, *M* = Na) used in dye industry. Their structures have been determined recently (Jacobs & Stahl, 2000). Only from lithium, the structures of the anhydrous compound (Yang *et al.*, 2001) as well as that of the dihydrate (Reuter & Bargon, 1997) are known. The last one crystallizes in the monoclinic space group *P*2_1_/*n* with [Sn(OH)_6_]^2-^ octahedra and [Li(µ_2_-OH)_3_(H_2_O)] tetrahedra linked in a three-dimensional way. By changing the crystallization conditions, we were able to isolate a new modification (in the following called β) of the dihydrate. This polymorph likewise crystallizes in the same space group and consists of the same building units as the known modification (in the following called α), but these primary units are linked two-dimensionally.

The asymmetric unit of the title compound (Fig. 1) consists of half a formula unit with the tin atom at a crystallographic centre of symmetry [Wyckoff letter 2*b*], and with all other atoms in general positions. Within the centrosymmetric hexahydroxidostannate(IV) anion, [Sn(OH)_6_]^2-^, the Sn—O bond lengths range from 2.0567 (11) to 2.0699 (11) Å [mean value: 2.064 (7) Å] and the O—Sn—O bond angles from 86.92 (4)° to 93.08 (4)° and are comparable with the corresponding values in the α-modification [*d*(Sn—O) = 2.053 (1) - 2.075 (1), mean value 2.060 (13) Å; O—Sn—O = 87.21 (4)° - 92.79 (4)°]. The lithium ion is tetrahedrally coordinated by three hydroxyl groups of three different hexahydroxidostannate(IV) anions and by one water molecule to form a [Li(µ_2_-OH)_3_(H_2_O)] unit. The Li—OH_hydroxyl_ bond lengths are in the range from 1.948 (3) Å to 1.965 (3) Å, mean value 1.958 (9) Å, whereas the bond to the water molecule is somewhat longer [2.030 (3) Å]. Although similar in constitution with the coordination polyhedron of Li in the α-modification, the Li—O bond lengths in the β-modification differ significantly. In the α-modification the Li—OH_hydroxyl_ bond lengths cover a larger range [1.932 (2) Å - 1.987 (2) Å] whereas the Li—H_2_O bond is considerably shorter [1.946 (3) Å].

In contrast to the previous modification, where [Sn(OH)_6_]-octahedra and [Li(OH)_3_(H_2_O)]-tetrahedra are linked three-dimensionally, they form in the new modification a two-dimensional layer structure (Fig. 2) parallel to (010). Within the almost planar layers, the [Sn(OH)_6_]-octahedra build up a hexagonal net with the tetrahedra in between, very similar to the situation of octahedral and tetrahedral voids in a close-packed layer. In summary, each octahedron is surrounded by six tetrahedra, with their apices in an alternating fashion up and down (Fig. 3).

In addition to the strong covalent and electrostatic interactions between cations, anions and water molecules, hydrogen bonds (Fig. 4) are important and fall into two categories: hydrogen bonds within the layers and those between the layers. The first ones are dominated by the water molecules that are coplanar with the layer plane and act as donors of two hydrogen bonds to two OH-groups of two different neighboring octahedra as well as acceptors of a hydrogen bond of an hydroxyl group of a third octahedron. The water molecules are also involved in the interlayer hydrogen bonds as acceptors resulting in an overall trigonal-bipyramidal coordination mode at the oxygen atom of the water molecule.

### Experimental

In a typical experiment, equimolar amounts of freshly prepared K_2_[Sn(OH)_6_] and LiNO_3_ were dissolved as far as possible in 15 ml H_2_O_2_ (15%_wt_). Undissolved reagents were removed by centrifugation before the solvent was allowed to evaporate slowly. Compact, colorless single crystals of the title compound were formed during some weeks.

### Refinement

Hydrogen atoms were clearly identified in difference Fourier syntheses. Their positions were refined with respect to a common O—H distance of 0.96 Å and for the water molecule an H—O—H angle of 104.9° before they were fixed and allowed to ride on the corresponding oxygen atoms. One common isotropic displacement factor was refined for all H-atoms.

### Crystal data

**Table:**

Li_2_[Sn(OH)_6_]·2H_2_O	*F*(000) = 260
*M**_r_* = 270.65	*D*_x_ = 2.661 Mg m^−^^3^
Monoclinic, *P*2_1_/*c*	Mo *K*α radiation, λ = 0.71073 Å
Hall symbol: -P 2ybc	Cell parameters from 9252 reflections
*a* = 6.1028 (2) Å	θ = 3.8–33.2°
*b* = 10.4708 (3) Å	µ = 3.78 mm^−^^1^
*c* = 6.0003 (2) Å	*T* = 100 K
β = 118.249 (1)°	Block, colourless
*V* = 337.76 (2) Å^3^	0.15 × 0.14 × 0.11 mm
*Z* = 2	

### Data collection

**Table:**

Bruker APEXII CCD diffractometer	986 independent reflections
Radiation source: fine-focus sealed tube	964 reflections with *I* > 2σ(*I*)
Graphite monochromator	*R*_int_ = 0.024
phi and ω scans	θ_max_ = 30.0°, θ_min_ = 3.8°
Absorption correction: multi-scan (*SADABS*; Bruker, 2009)	*h* = −8→8
*T*_min_ = 0.601, *T*_max_ = 0.681	*k* = −14→14
11484 measured reflections	*l* = −8→8

### Refinement

**Table:**

Refinement on *F*^2^	Secondary atom site location: difference Fourier map
Least-squares matrix: full	Hydrogen site location: inferred from neighbouring sites
*R*[*F*^2^ > 2σ(*F*^2^)] = 0.013	H-atom parameters constrained
*wR*(*F*^2^) = 0.032	*w* = 1/[σ^2^(*F*_o_^2^) + (0.0111*P*)^2^ + 0.5571*P*] where *P* = (*F*_o_^2^ + 2*F*_c_^2^)/3
*S* = 1.09	(Δ/σ)_max_ < 0.001
986 reflections	Δρ_max_ = 0.36 e Å^−^^3^
54 parameters	Δρ_min_ = −0.39 e Å^−^^3^
0 restraints	Extinction correction: *SHELXL97* (Sheldrick, 2008), Fc^*^=kFc[1+0.001xFc^2^λ^3^/sin(2θ)]^-1/4^
Primary atom site location: structure-invariant direct methods	Extinction coefficient: 0.0094 (10)

### Special details

**Table:**

Geometry. All e.s.d.'s (except the e.s.d. in the dihedral angle between two l.s. planes) are estimated using the full covariance matrix. The cell e.s.d.'s are taken into account individually in the estimation of e.s.d.'s in distances, angles and torsion angles; correlations between e.s.d.'s in cell parameters are only used when they are defined by crystal symmetry. An approximate (isotropic) treatment of cell e.s.d.'s is used for estimating e.s.d.'s involving l.s. planes.
Refinement. A suitable single-crystal was selected under a polarization microscope and mounted on a 50 µm MicroMesh MiTeGen Micromount^TM^ using FROMBLIN Y perfluoropolyether (LVAC 16/6, Aldrich).Refinement of *F*^2^ against ALL reflections. The weighted *R*-factor *wR* and goodness of fit *S* are based on *F*^2^, conventional *R*-factors *R* are based on *F*, with *F* set to zero for negative *F*^2^. The threshold expression of *F*^2^ > σ(*F*^2^) is used only for calculating *R*-factors(gt) *etc*. and is not relevant to the choice of reflections for refinement. *R*-factors based on *F*^2^ are statistically about twice as large as those based on *F*, and *R*- factors based on ALL data will be even larger.

### Fractional atomic coordinates and isotropic or equivalent isotropic displacement parameters (Å^2^)

**Table:**

	*x*	*y*	*z*	*U*_iso_*/*U*_eq_	
Sn1	0.5000	0.5000	0.5000	0.00669 (7)	
O1	0.5924 (2)	0.61350 (11)	0.2773 (2)	0.0092 (2)	
H1	0.4344	0.6208	0.1278	0.045 (4)*	
O2	0.2930 (2)	0.38228 (11)	0.1944 (2)	0.0092 (2)	
H2	0.3438	0.2947	0.2061	0.045 (4)*	
O3	0.1780 (2)	0.60408 (11)	0.3926 (2)	0.0088 (2)	
H3	0.1965	0.6930	0.3659	0.045 (4)*	
Li1	0.8284 (5)	0.5657 (3)	0.1561 (6)	0.0123 (5)	
O4	0.8497 (2)	0.37229 (11)	0.1795 (2)	0.0101 (2)	
H41	0.8643	0.3771	0.3458	0.045 (4)*	
H42	1.0175	0.3651	0.2071	0.045 (4)*	

### Atomic displacement parameters (Å^2^)

**Table:**

	*U*^11^	*U*^22^	*U*^33^	*U*^12^	*U*^13^	*U*^23^
Sn1	0.00660 (8)	0.00674 (9)	0.00670 (9)	0.00001 (4)	0.00314 (6)	0.00009 (5)
O1	0.0091 (5)	0.0103 (5)	0.0081 (5)	−0.0009 (4)	0.0039 (4)	0.0007 (4)
O2	0.0096 (5)	0.0088 (5)	0.0084 (5)	0.0005 (4)	0.0035 (4)	−0.0003 (4)
O3	0.0085 (5)	0.0075 (5)	0.0107 (5)	0.0006 (4)	0.0047 (4)	0.0006 (4)
Li1	0.0136 (12)	0.0121 (13)	0.0117 (13)	−0.0001 (10)	0.0063 (11)	−0.0006 (10)
O4	0.0094 (5)	0.0116 (5)	0.0096 (5)	−0.0002 (4)	0.0048 (4)	−0.0004 (4)

### Geometric parameters (Å, º)

**Table:**

Sn1—O1^i^	2.0567 (11)	O2—H2	0.9600
Sn1—O1	2.0567 (11)	O3—Li1^iii^	1.965 (3)
Sn1—O3^i^	2.0654 (11)	O3—H3	0.9600
Sn1—O3	2.0654 (11)	Li1—O2^ii^	1.948 (3)
Sn1—O2^i^	2.0699 (11)	Li1—O3^iv^	1.965 (3)
Sn1—O2	2.0699 (11)	Li1—O4	2.030 (3)
O1—Li1	1.961 (3)	O4—H41	0.9600
O1—H1	0.9600	O4—H42	0.9600
O2—Li1^ii^	1.948 (3)		
			
O1^i^—Sn1—O1	180.0	Sn1—O1—H1	101.0
O1^i^—Sn1—O3^i^	90.31 (4)	Li1^ii^—O2—Sn1	123.42 (11)
O1—Sn1—O3^i^	89.69 (4)	Li1^ii^—O2—H2	106.9
O1^i^—Sn1—O3	89.69 (4)	Sn1—O2—H2	117.6
O1—Sn1—O3	90.31 (4)	Li1^iii^—O3—Sn1	131.67 (10)
O3^i^—Sn1—O3	180.0	Li1^iii^—O3—H3	104.5
O1^i^—Sn1—O2^i^	90.49 (4)	Sn1—O3—H3	113.2
O1—Sn1—O2^i^	89.51 (4)	O2^ii^—Li1—O1	110.99 (15)
O3^i^—Sn1—O2^i^	86.92 (4)	O2^ii^—Li1—O3^iv^	116.79 (15)
O3—Sn1—O2^i^	93.08 (4)	O1—Li1—O3^iv^	114.65 (15)
O1^i^—Sn1—O2	89.51 (4)	O2^ii^—Li1—O4	109.44 (15)
O1—Sn1—O2	90.49 (4)	O1—Li1—O4	105.27 (14)
O3^i^—Sn1—O2	93.08 (4)	O3^iv^—Li1—O4	98.16 (13)
O3—Sn1—O2	86.92 (4)	Li1—O4—H41	89.5
O2^i^—Sn1—O2	180.0	Li1—O4—H42	96.8
Li1—O1—Sn1	124.68 (10)	H41—O4—H42	105.0
Li1—O1—H1	105.2		

Symmetry codes: (i) −*x*+1, −*y*+1, −*z*+1; (ii) −*x*+1, −*y*+1, −*z*; (iii) *x*−1, *y*, *z*; (iv) *x*+1, *y*, *z*.

### Hydrogen-bond geometry (Å, º)

**Table:**

*D*—H···*A*	*D*—H	H···*A*	*D*···*A*	*D*—H···*A*
O1—H1···O4^ii^	0.96	1.84	2.7955 (16)	173
O2—H2···O1^v^	0.96	1.93	2.8853 (16)	173
O3—H3···O4^vi^	0.96	1.90	2.8343 (16)	164
O4—H41···O3^i^	0.96	1.72	2.6634 (16)	167
O4—H42···O2^iv^	0.96	1.73	2.6662 (16)	165

Symmetry codes: (i) −*x*+1, −*y*+1, −*z*+1; (ii) −*x*+1, −*y*+1, −*z*; (iv) *x*+1, *y*, *z*; (v) −*x*+1, *y*−1/2, −*z*+1/2; (vi) −*x*+1, *y*+1/2, −*z*+1/2.
